# Supplementary material for: Dynamic Organizational Strategies of Multidomain Glycosyltransferases Revealed by High-Speed AFM and Solution Biophysics
Source: Int J Mol Sci. 2026 Jul 19;27(14):6423. doi: 10.3390/ijms27146423 (PMC13411425; doi:10.3390/ijms27146423)
Supplement: Supplementary file 1 [file ijms-27-06423-s001.zip › Supplementary data.pdf]

Supplementary Figure S1

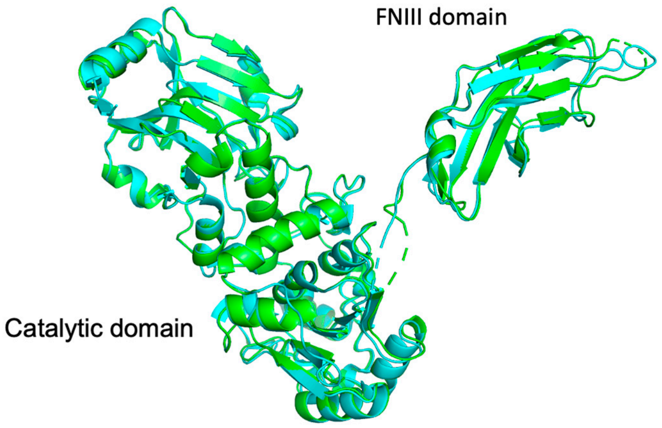

Supplementary Figure S1. Structural comparison of POMGNT2 with the previously reported structure.

Superposition of the crystal structure of POMGNT2 determined in this study (green) (PDB code: 8KB7) with the previously reported POMGNT2 structure (cyan) (PDB code: 7E9J). POMGNT2 consists of an N-terminal catalytic domain and a C-terminal FnIII domain, which adopt a similar overall arrangement to that observed in the previous structure. Crystallographic data collection and refinement statistics are summarized in Supplementary Table S1.

Supplementary Figure S2

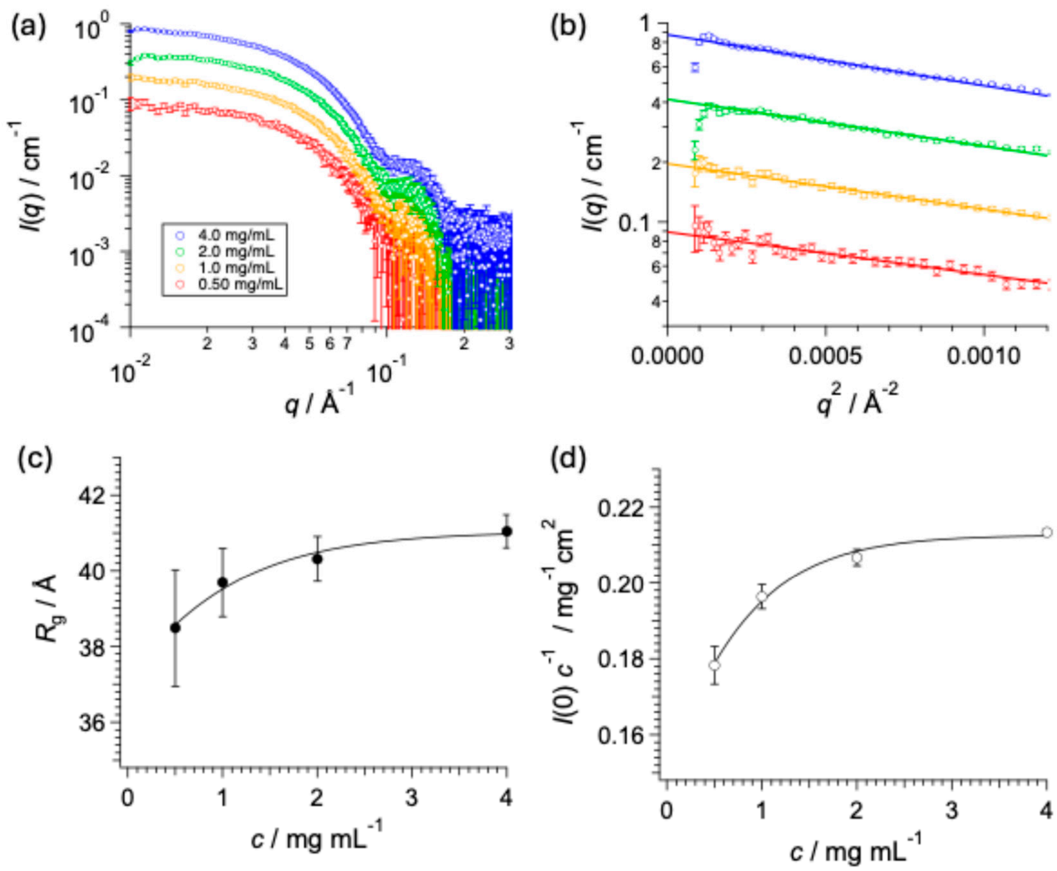

Supplementary Figure S2. SAXS analysis of K4CP at different protein concentrations.

(a) SAXS profiles of K4CP were recorded at 0.5 (red circles), 1.0 (yellow circles), 2.0 (green circles), and 4.0 mg/mL (blue circles). (b) Guinier plots of the corresponding scattering intensities. Solid lines represent the mean square fitting by Guinier function. (c) Concentration dependence of the radius of gyrations R_g offered by the Guinier analysis. Solid curve represent the fitting curve by Sigmoidal function for eye guide. (d) Concentration dependence of the forward scattering intensity $I(0)$ offered by the Guinier analysis. Solid curve represent the fitting curve by Sigmoidal function for eye guide. Both R_g and $I(0)$ increased with the K4CP concentration and reached an apparent plateau at 4.0 mg/mL. This concentration dependentence indicates an increasing fraction of dimeric species, suggesting that the scattering contribution at 4.0 mg/mL is dominated by the dimer.

Supplementary Figure S3.

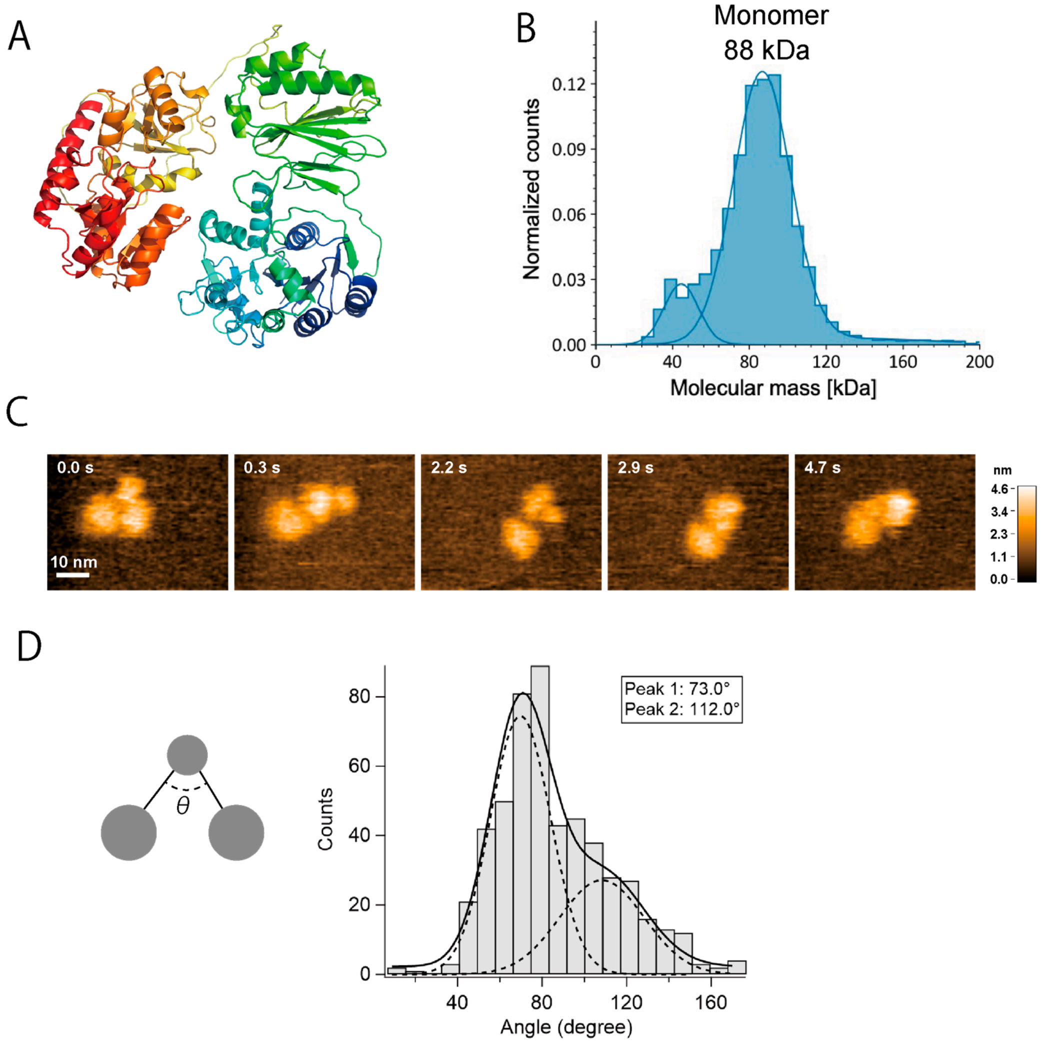

Supplementary Figure S3. Structural and dynamic analysis of the giant-virus-derived L137 protein by mass photometry and HS-AFM.

(A) AF3-predicted structural model of giant-virus-derived L137 shown in a monomeric arrangement. (B) Mass photometry analysis of purified L137. The major population corresponded to monomeric L137. (C) Representative time-lapse HS-AFM images of L137 particles. Three lobular densities were repeatedly observed within individual particles, and their relative positions changed over time, indicating conformational flexibility. The time-lapse sequence corresponding to these HS-AFM images is shown in Video S4. The two larger lobes likely correspond to the two domains of the L137 monomer, whereas the smaller central density represents an additional globular density between these domains, consistent with the AF3-predicted monomeric model. Imaging conditions: 93 × 64 pixels, 56 × 42 nm^2 scan area, 100 ms per frame; scale bar, 10 nm; height scale, 0–4.6 nm. (D) Schematic representation of the three-lobed particle model used to quantify inter-lobe angles from HS-AFM

images, together with the distribution of measured inter-lobe angles. The histogram was fitted with Gaussian components, revealing a major population centered at approximately 73° and a minor population centered at approximately 112° (n = 520 measurements from 5 particles). These results indicate that L137 adopts a flexible three-lobed architecture in solution, consistent with the AF3-predicted monomeric structural model.

Supplementary Table S1. Data collection and refinement statistics for UDP/Man-bound POMGNT2

	Native	K_2PtCl_4
**Crystallographic data**		
Space group	$P2_12_12_1$	$P2_12_12_1$
Unit cell *a*/*b*/*c* (Å)	145.0/150.0/191.5	145.4/151.2/191.7
**Data processing statistics**		
Beam line	SPring-8 BL44XU	SPring-8 BL44XU
Wavelength (Å)	0.9000	1.0717
Resolution (Å)	50-2.80 (2.97–2.80)	48.79-3.71(3.84–3.71)
Total/unique reflections	705,396/197,873	1,214,082/45,325
Completeness (%)	99.7 (99.3)	99.6 (96.3)
R_{merge} (%)	6.2 (91.8)	24.8 (184.6)
$CC_{1/2}$	99.9 (59.6)	99.9 (75.9)
$I / \sigma (I)$	13.5 (1.2)	12.6 (1.8)
Multiplicity	3.6 (3.4)	26.8 (18.2)
**Refinement statistics**		
Resolution (Å)	47.92-2.80	
R_{work} / R_{free} (%)	19.1/22.8	
R.m.s. deviations from ideal		
Bond lengths (Å)	0.008	
Bond angles (°)	1.63	
Ramachandran plot (%)		
Favored	93.9	
Allowed	5.5	
Disallowed	0.6	
Number of atoms		
Protein atoms (A/B/C/D)	4192/4121/4163/4157	
UDP	25/25/25/25	
Mannose	12/12/12/12	
Average *B* factors ($Å^2$)		
Protein atoms	88.1/90.7/95.2/102.3	
UDP	104.7/153.5/133.7/147.5	
Mannose	97.7/111.5/108.5/116.6	

Video S1. HS-AFM imaging of POMGNT2 molecules in solution.

Time-lapse high-speed atomic force microscopy images showing the dynamic behavior of POMGNT2 molecules on the mica surface. The elapsed time is shown in the movie. Scale bar, 10 nm.

Video S2. HS-AFM imaging of LARGE1 molecules in solution.

Time-lapse high-speed atomic force microscopy images showing the dynamic behavior of LARGE1 molecules on the mica surface. The elapsed time is shown in the movie. Scale bar, 5 nm.

Video S3. HS-AFM imaging of K4CP molecules in solution.

Time-lapse high-speed atomic force microscopy images showing the dynamic behavior of K4CP molecules on the mica surface. The elapsed time is shown in the movie. Scale bar, 10 nm.

Video S4. HS-AFM imaging of L137 molecules in solution.

Time-lapse high-speed atomic force microscopy images showing the dynamic behavior of L137 molecules on the mica surface. The elapsed time is shown in the movie. Scale bar, 10 nm.
